# Effects of Intergenerational Care Behavior on Residents’ Nutrition Intake—Descriptive Statistical Analysis of Rural China Survey 2010–2014

**DOI:** 10.3390/foods12010118

**Published:** 2022-12-26

**Authors:** Huaquan Zhang, Fan Yang, Martinson Ankrah Twumasi, Abbas Ali Chandio, Ghulam Raza Sargani

**Affiliations:** College of Economics, Sichuan Agricultural University, Chengdu 611130, China

**Keywords:** nutritional intake, intergenerational care, rural China, fixed effect

## Abstract

Nutritional status plays an indispensable role in enhancing human capital and preventing the return to poverty. In the context of the three-child policy and the aging society in China, intergenerational care will inevitably become a core component of family support. Thus, this paper evaluates the impacts of intergenerational care behavior on nutritional intake in Chinese rural residents from the perspective of household consumption. The study’s data is from the China Family Panel Studies (CFPS) (2010–2014), and, the fixed effect model and analyses are applied to achieve the study’s objective. The results reveal that rural residents with intergenerational family care behavior have significantly higher food diversity. Thus, Chinese rural residents with intergenerational care consumed 22.4% more food. A possible mechanism is that intergenerational care improves young mothers’ labor participation and income, thus optimizing their dietary structure. Moreover, there was heterogeneity concerning the nutrition intake among rural residents in different geographic and family net income groups. Finally, the robust check is consistent with the baseline regression results. In summary, governments should fully affirm the elderly contribution to families to give full play to the elderly family benefit maximization.

## 1. Introduction

According to the “Healthy China 2030” plan, the Chinese government has emphasized the strategic role of health in China’s development [1]. At the same time, the previous literature has shown that dietary intake is strongly associated with health, and a low-quality diet intake is one of the critical causes of health risks and chronic diseases [2]. Thus, food diversity and consumption contribute to the well-being of individuals. For example, studies including [3,4] have shown that lack of food diversity among households leads to undernutrition, obesity, and overweight, negatively affecting household welfare. In addition, households unable to diversify their food consumption are likely to experience micronutrient deficiency, an enhancer of poor well-being. Again, evidence also indicates that poor physical growth, impaired cognition, morbidity, and mortality are highly connected with poor food diversity [4].

As of 2020, China, the biggest developing country, has achieved comprehensive poverty alleviation with a rapidly developed economy. This great achievement can significantly influence residents’ nutritional status and diet structure in several ways. However, there still exists a greater risk of returning to poverty, and the main cause of returning to poverty is health problems. In China, the rural population still accounts for 36.11%, and although the dietary quality of rural residents in China has been significantly improved, the economic development levels and food consumption groups between urban and rural areas are still unequal. Particularly, the incidence of malnutrition, suboptimal health, and mortality is prevalent among rural residents because of the low individual incomes, poor diet knowledge and infrastructure, and little access to markets [3]. It is widely recognized that diet variety and quality tend to be associated with higher nutritional adequacy [4]; thus, inadequate nutrition significantly constrains good health among farmers, affecting their human capital. Moreover, farmers’ ability or opportunity to create income is damaged by low health, thereby causing economic and welfare loss and opening the opportunity for individuals to fall into and stay in poverty [5]. Improving nutritional status is an essential component of achieving the Sustainable Development Goals 2 (SDG 2) agenda since it is indispensable in enhancing human capital and reducing poverty [6]. Therefore, it is imperative to pay attention to assessing the main factors related to the nutritional intake of farmers and empirically test the possible paths of nutrition improvement heterogeneously among farmers to promote social equity.

Understanding the determinants of food intake to improve an individual’s nutrition is an important research area [7]. A significant body of existing literature has been published to reveal the factors affecting nutritional intake, including urbanization [8], internet [6], female empowerment [9], income [10], farmers’ production structure, and external market conditions [5], microcredit [11], food accessibility [12], educational attainment [13], mobile phones [14], etc. To the best of our knowledge, the previous literature seldom investigates nutritional intake from the perspective of household composition and population structure on food consumption. Redman [15] found that American families with preschoolers and older women ate out less than other families. Again, according to the National Bureau of Statistics in China, the country’s age structure is changing with a longer life expectancy, which means that China’s elderly human capital is continuously rising [16]. Statistics show that there are more than 260 million people over the age of 60 as of 2020, accounting for 18.7% of the total population. The proportion of the aging population is expected to grow to 34% by 2050 [17]. Increasing life expectancy and substantially reduced child mortality means that the multiple generations will exist in large quantities, and grandparents may often provide care and assistance to their grandchildren [18,19]. Thus, contrary to previous studies, we mainly explored the relationship between intergenerational household care and nutritional intake to more intuitively assess the effects of intergenerational household care on well-being.

According to the cultural values and family norms in other parts of the world, such as Europe and America, grandparents are not culturally expected to have the custodial responsibility of caring for grandchildren. However, in contemporary rural and urban China, grandparents caring for grandchildren is common for many traditional families [20]. More specially, grandparents usually provide downward support and use their saved economic or social resources to help their children relieve burdens, including divorce, poverty, substance abuse, mental health problems, and incarceration, due to the traditional Chinese “parental system” and the philosophy of Confucianism influence [21]. Even if elderly parents do not live with their children, they still play a very important role in their children’s lives. In the past, the identity of the elderly group in the family was usually “caregiver”, but with the development of social pension insurance and proper healthcare environment, the physical fitness and the life expectancy of the elderly group have increased significantly, meaning more children have living grandparents [22]. At the same time, under the background of China’s comprehensive three-child policy, the shortage of childcare resources and the imperfect public childcare service system make it impossible for families to meet their childcare needs. A study found that filial piety reflects the norm of reciprocity, and taking care of grandchildren can be regarded as a bargaining strategy to ensure their elderly support when families face heavy child-raising pressures [23]. Another study showed that the role of grandparents begins to transform from the past “uninvolved” to “occasional helper” or “long-term surrogate parent” [24]. In addition, mainly grandmothers serve as the primary substitute for parents and social parenting institutions, providing informal childcare resources to relieve the parenting pressure of the biological parents [25,26]. Therefore, in this context, understanding the role of grandparents in providing informal childcare will indirectly reduce the cost of family child-rearing and has a far-reaching external effect on the family labor participation rate. Freeing the mother from the burden of housework and thus having more time to participate in economic activities ultimately benefits the entire extended family [20].

The present paper contributes to analyzing whether Chinese families, through intergenerational care, can improve nutritional intake. The remaining parts of this paper are organized as follows: In the next section, we put forward the theoretical framework and research hypothesis, and in Section 3, we introduce the data source and model set, while showing the analysis of the empirical results in Section 4. Finally, Section 5 reports the conclusion of our empirical results and a brief discussion of our study’s policy implications and limitations.

## 2. Theoretical Framework and Hypotheses

Among the many factors affecting food consumption, income plays an irreplaceable role in the dietary structure. Income growth will increase food diversity consumption and lead to more nutritionally diverse diets to a certain extent [27]. However, in China, the social care resources for children are becoming short and expensive. Rising housing prices and living costs make it difficult for families to raise children, and there is a greater burden on young parents’ labor participation. In particular, influenced by traditional gender concepts and female reproductive roles, young women tend to be the main bearers of family care responsibilities, and they will spend more time and energy on family care, resulting in a lower labor participation rate of young women. However, in the context of China’s aging, from the perspective of maximizing the whole family’s well-being, the elderly could be interpreted as an adaptive family strategy to take care of their grandchildren by alleviating young mothers from heavy household chores and reducing their family burden, thus promoting the labor supply of young mothers to pursue economic opportunities [20], or other grandparents may do so in exchange to ensure old age support [19]. As young women’s labor participation rate increases, it will inevitably lead to an income increase and the rationalized distribution of family wealth resources. When food consumption is no longer constrained by income, residents will inevitably increase the types of food consumption to improve their dietary health. The following hypotheses are proposed in this article:

**Hypothesis** **1** **(H1):** *Intergenerational care can significantly improve the young female’s income*.

**Hypothesis** **2** **(H2):** *The increase in young women’s personal income is conducive to family diet diversity and nutrient intake*.

Nutrition and health play an important promotion of human capital. Here, we showed that grandparents play a role in household diet diversity and nutritional intake. Thus, on the one hand, in the absence of necessary social children care services, grandparents who may have more extra time than younger parents tend to provide informal care and housework such as meticulously preparing daily household meals. On the other hand, the grandparents’ intergenerational care will provide their grandchildren with economic and social resources. To a certain extent, they will play a replacement role in social care institutions, reduce family parenting expenditures, improve family financial conditions, and promote the reasonable allocation of household wealth resources, which will be used for various diet consumption and promote diet diversity and nutritional intake of the family. The following assumption is proposed in this article:

**Hypothesis** **3** **(H3):** *Intergenerational care behavior will promote food consumption and nutritional intake*.

## 3. Materials and Methods

### 3.1. Data Collection

The data of this article come from Peking University’s Social Science Survey named China Family Panel Studies (CFPS), which is a biannual longitudinal survey of Chinese including communities, families, and individual levels, aiming to reflect the changes in China’s society, population, education, and health. Since 2010, CFPS has conducted a continuous traceable survey of more than 25 provinces with a target sample size of 16,000 households in mainland China every two years. Although the CFPS database has been updated to 2020, the detailed food intake (i.e., diet over the past week) information of respondents is not available in CFPS 2016–2020 yet. This means that this study can only utilize the CFPS data up to 2014.

Thus, we estimated the intergenerational care effects on household food security and nutrition using the 2010–2014 survey as research samples with family, children, and individual databases. In this paper, we focus on intergeneration care on food diversity; based on the study design, we excluded all samples that did not have children in rural families. Furthermore, in terms of age, taking into account the key feature of the ability to provide intergenerational care within the family, men aged 22–59 years old and women aged 20–49 years old were selected as the baseline study subjects. Finally, a total of 20904 respondents screened from 25 provinces (i.e., Hebei, Jilin, Fujian, Shandong, Henan, and Yunnan) remained.

### 3.2. Methods

#### 3.2.1. Independent Variables

The first core keyword of China’s “2021 Dietary Guidelines” is food diversity. Nutritionists usually consider that a single food cannot provide various nutrients needed by the human body, but more variety of foods can provide rich nutrients. Thus, dietary diversity scores count the number of different food groups consumed over a specified period of time and are a widely used comprehensive tool to assess food security and dietary adequacy [14,28]. The advantage of dietary diversity scores is that the calculation is simple, as long as the total score if for the different food consumption over the week without considering the specific days that households consumed those foods [29]. As a result, our key independent variable measures the degree of household food consumption and dietary practices to analyze the relationship between intergenerational care and the dietary diversity of rural residents.

CFPS survey obtains data on individuals who consumed 9 major categories of food in the past week, so the food diversity scores were consumed specific food groups by any household member in the given seven-day period. A score closer to 9 implies high dietary diversity intake. The 9 food groups considered are as follows: meat; fish and other aquatic products; fresh vegetables and fruits; dairy products; soy products; eggs; cereals; pickled foods (such as squeezed vegetables, soy sauce tofu); puffed/fried foods (e.g., potato chips, fritters). However, the CFPS2010 obtains data on individuals who consumed 9 major categories of food in the past month, and we regard it as the 9 types of foods in the past week.

#### 3.2.2. Core Dependent and Mediator Variables

The main dependent variable of interest is intergenerational care, which refers to the act of grandparents caring for grandchildren without compensation for altruistic motives or other reasons. It is captured through a dummy variable generated based on the question, “Who cares for the child during the day and at night”, excluding families without children, when respondents answered that they were cared for by “the child’s grandparents/grandmothers”, “intergenerational care = 1”; conversely, “alternate care = 0”.

In terms of mediator variables, we are particularly interested in the household’s young female personal income. Female personal income is equal to the sum of the various incomes such as agricultural and nonagricultural income. To avoid the effect of outliers on the results, responders with a total personal income lower than 5% or higher than 95% of the sample were excluded, and then we performed logarithmic applications.

#### 3.2.3. Control Variables

According to previous studies on nutrition estimation, several demographic variables and household characteristics were used as control variables in the multiple regressions. Numerous studies have investigated the responders’ education factor of food diversity. Ji et al. [30] pointed out that the high educational level of the mother is the protective factor of healthy dietary behavior, so the respondent’s education and the education level of the grandparents are included. The body needs different nutrients at different ages, so age is often seen as a variable that affects diet diversity. Furthermore, marriage status and a family’s child numbers are also used as control variables. In the aspect of household characteristics, food accessibility is an essential factor that may affect food consumption. Chinnadurai et al. [31] suggest that having access to markets may have more potent effects on dietary diversity, so we control the distance to the nearest market town. Rural areas mainly consume self-produced food, which directly influences the whole family’s dietary diversity and nutrition intake [32]. Considering that land rent may impact household food supply and thus food consumption, whether the land is rent was included as a control variable. Moreover, Ruel et al. [33] find that household size significantly increases the expenditure on breakfast consumption; that is, household size is included. Similarly, other household characteristics were also included. A series of papers has empirically shown that there is a strong link between family income and dietary diversity. Family income is recognized to play a significant role in dietary diversity; higher-income families exerted a positive effect on food diversity [34,35], and people with higher family income appear to have a healthier diet [35,36]. Thus, instead of using individual income, family net income is used in the estimation because food consumption and nutrition-related decisions are normally made at the household level, so we refer to family net income as a control variable, but the distance to the nearest market town and family net income showed wide variation among households. Thus, to avoid heteroscedasticity, logarithmic transformation was applied, respectively.

#### 3.2.4. Model Specification

(1) Benchmark regression

We use three years of unbalanced panel data to explore whether intergenerational care behavior can help residents’ dietary diversity. Our outcome variables are the different measures of dietary diversity as described above, so the fixed effect model (FE) is adopted in this paper, which can eliminate the influence of unobservable variables that do not change over time on the dependent variable through the difference method and can also overcome the endogeneity problem of potential omitted variables. We estimate a fixed effect (FE) model with the generic form of

(1) $${\mathrm{DDS}}_{\mathrm{it}}=\beta_{0}+\beta_{1}{\mathrm{care}}_{\mathrm{it}}+{\gamma X}_{\mathrm{it}}+\varepsilon_{\mathrm{it}}$$

In the equation, the variable ${\mathrm{DDS}}_{\mathrm{it}}$ is the measure of dietary diversity score, which represents the type of food consumption at time t, and the core explanatory variable ${\mathrm{care}}_{\mathrm{it}}$ represents the intergenerational care of resident i at time t. Variable $X_{\mathrm{it}}$ is a set of characteristics of other microlevel factors of individual and household characteristics associated with resident i at time t, including age, education level, marital status, distance, land rent, etc. $\varepsilon_{\mathrm{it}}$ is the random disturbance term, and γ, and $\beta_{0}$ are our parameter of interest.

(2) Analyzing possible mechanisms.

In order to explore the possible influence channels between intergenerational care and family dietary diversity, this paper further introduced the mediation effect test method proposed by Wen et al. [37] to further analyze the influencing mechanism. We estimate the following models to gain further insights into possible mechanisms:

(2) $$M_{\mathrm{it}}=\alpha_{0}+\alpha_{1}{\mathrm{care}}_{\mathrm{it}}+{\gamma X}_{\mathrm{it}}+\varepsilon_{\mathrm{it}}$$

(3) $${\mathrm{DDS}}_{\mathrm{it}}=\gamma_{0}+\gamma_{1}{\mathrm{care}}_{\mathrm{it}}+\gamma_{3}M_{\mathrm{it}}+{\gamma X}_{\mathrm{it}}+\varepsilon_{\mathrm{it}}$$

where ${\mathrm{DDS}}_{\mathrm{it}}$ is the household dietary diversity score; $M_{\mathrm{it}}$ is the mediating variable, including female personal income; $\beta_{1}$ represents the total effect of intergenerational care on family dietary diversity; $\gamma_{1}$ is the direct utility of intergenerational care on the explained variable; $\alpha_{1}$, $\gamma_{3}$ is the magnitude of the mediation utility. $\gamma_{0}\;\mathrm{and}\;\alpha_{0}$ are our parameter of interest. On the basis of the significant coefficient of $\beta_{1}$, if both $\alpha_{1}$ and $\gamma_{3}$ are significant, we could cautiously conclude that the mediating effect is significant, and the effects of intergenerational care on nutrition are mainly channeled through female personal income. If the bootstrap statistic is constructed to test the significance of its mediating utility, and only the bootstrap statistic is significant, there is really a mediating effect.

## 4. Results and Discussions

### 4.1. Descriptive Statistics

Table 1 shows the descriptive statistics for the main variables used in the analysis of this paper. As shown in a period of seven days, the average food diversity was 4.907, indicating a variety of foods eaten last week; meanwhile, the independent variable of intergenerational care was 0.554, which means there are 55.4% of households with intergenerational care behavior. The individual characteristics of the households indicate that the average age of the respondents was 42.11 years old, mainly middle-aged rural residents. On average, most of the rural adults were married with a mean score of 0.972, but the average education year of rural adults was 6.483, which means the education level at primary school accounted for half of the study population. Furthermore, based on family control variables, it can be seen that the average family size was 4.674, and only 8.0% of rural households rented out their land. After logarithmically processing, the mean values of distance and family net income were 2.881and 10.147, respectively. Finally, it is found that the average education year of grandparents is 5.214.

### 4.2. Baseline

The estimation impact of intergenerational care on food diversity for the unbalanced panel data is presented in Table 2. In column (1), we first adopt the panel data fixed effect model, which found that elderly parents’ intergenerational care could improve the dietary diversity in rural areas by 22.4%, significant at the 1% level. In contrast, the random effect model (2) was used for comparison and robust check. In general, the result from the RE estimator is largely consistent with the results from the FE estimator. The result also showed that intergenerational care has a positive and significant connection with the intake of food diversity, which implies that intergenerational care by grandparents will increase dietary richness by one more food item. What is more, we analyzed whether and to what extent individual and family characteristics have an impact on food diversity. We find, as shown in column (1), that as respondent education increases, the rural residents’ diet diversity will increase by 6.5%. Furthermore, to assess whether the land rent out has a correlation to their food intake, the results of the panel data FE model estimation show that respondents with the land rent out have a positive effect on their nutritional intake by 2.5%. Furthermore, as we expected, with a family net income increase by 1%, the rural residents’ diet diversity will significantly increase by 12.1% because there is a greater chance that their income will translate into diverse diets [38]. As Ren et al. recognizes, high-income groups are more likely to obtain information about healthy diets and transform diet knowledge into action [39]. On the contrary, the poor represent the most harmed population by the diverse diet since they consume a typical, monotonous diet based on starchy staple foods containing little or no animal products, dairy products, and fewer fruits and vegetables, leading to a variety of nutrient deficiency [35]. Similarly, grandparents’ education year is associated with higher consumption of food. This implies that the level of the grandparents’ education has a significant positive role in household decision-making. However, the coefficient of distance to the nearest market town and family size in the estimation for food intake is negative. Food accessibility is an essential factor affecting food consumption. Hence, the farther the distance to the nearest market, the harder the accessibility of food will be. In other words, a far distance to the town market may reduce the level of food accessibility. Finally, the larger the size of the family, the heavier its family pressure burden; thus, the quality of life of the family will decline overall, which will affect the diet diversity.

What is more, the fixed effect model cannot solve the indigenousness caused by reverse causality and endogeneity due to omitted variables that change over time. Therefore, in column (3) we try to take an instrumental variable approach to eliminate the endogeneity problem. According to the selection conditions of the instrumental variables, “Whether grandparents are alive?” was selected as the instrumental variable in this study. The specific results show that when considering the endogeneity of intergenerational care, under other conditions unchanged, having intergenerational care will increase dietary diversity, which is statistically significant at the 1% level and consistent with our theoretical hypothesis (3).

### 4.3. Mechanism Analysis

In recent years, a growing body of scholars began to question the validity and rationality of the stepwise regression mediation effect test method and proposed a more reliable mediation effect test procedure and bootstrap method [40,41]. Thus, on the basis of confirming that elderly parents’ intergenerational care can promote diet diversity, we use the bootstrap method, which was extracted 2000 times to gain further insights into possible mechanisms of intergenerational care, and test the mediating effect of female labor participation and labor income, respectively, as the results show in Table 3. First of all, the indirect and direct effects of female personal income were (0.001,0.024) and (−0.027, 0.279) with 95% confidence intervals, respectively, but the direct effects of the confidence interval include 0, indicating that a completely mediating effect exists. Thus, intergenerational care can promote young women’s personal income, and with the increase in young women’s personal income, dietary diversity will be promoted. It is suggested that individual female income is the main channel through which intergenerational care affects nutritional intake; that is, the higher the income of a female, the more reasonable the diet. Therefore, hypotheses (1) and (2) are all confirmed.

### 4.4. Heterogeneity Effect

Since China is a vast country with a huge population, considering that there are relatively large differences among various rural residents, the impact of intergenerational care may likewise show heterogeneity in diet diversity. To further analyze the heterogeneity of results, this study first divided the sample by region and family net income to investigate the impacts of intergenerational care on the nutritional intake of rural residents. Firstly, according to China’s geographical differences, there are significant differences in economic development and social resources between the eastern and western regions, so the samples were divided into eastern, central, and western regions to verify whether the impact of intergenerational care effects is different between residents who live in different geographical. Regression results in Table 4 show that the impact of intergenerational care behavior on family dietary diversity in western China is positive but not significant. The reason may be interpreted that the western rural area has a backward market economy and less food diversity; elderly parents also usually work busy farming jobs for life, so they do not have the extra time for elaborate preparations for daily diet. Moreover, the diet acknowledgment in western China is also more backward; thus, the influence of intergenerational care on family diet diversity is not significant. However, the effect of intergenerational care behavior on family dietary diversity in the central region and eastern region were significantly positive, which was consistent with the regression results of the total sample. It can be seen that the regression results in different regions are quite different, and there are also some differences with the basic regression. In line with Zhao et al., the different personal reactions seemed to govern the social demographic statistics groups [42]. Therefore, comparing the groups with the whole sample indicated that the regression in different areas is quite different among the groups and there are also some differences with the whole sample basic regression.

Secondly, we compared the heterogeneity of the impact of intergenerational care between the different family’s net income in rural residents. We divided the family net income level into two groups, and the empirical results in Table 5 report the impact of differences in the per capita household income on diet diversity. As can be seen from Table 5, the intergenerational care activities all improved the family diet, which confirms the robustness of the baseline regression conclusion from one side. However, considering the difference in the degree of family net income, column (1) shows that intergenerational care has a significant influence on the family diet in the category of lower-level family net income; in contrast, column (2) indicates that the dietary improvement effect of those with a higher family net income level was insignificant. The differences in outcome can be explained that households with higher per capita income have more money to spend on improving the family diet, while households with a lower family net income tend to limit their dietary consumption so intergenerational care behavior can relieve the rearing cost of their families and finally alleviate the family financial pressure to promote a reasonable dietary structure of the family.

### 4.5. Robustness Check

In order to test the reliability of the basic regression results, this article mainly conducted robust tests from the following two aspects: First of all, in reference to the research of Sun and Zhang [43], due to the strong correlation between generational care and respondent age, we added the quadratic age variables to eliminate the possible nonlinear estimation bias caused by age. In addition, we changed independent variables to some other healthy dietary variables. Considering that the basic regression included two types of unhealthy diets (pickled food and fried foods), eating only the other seven types of foods is healthier than eating all categories of foods. Therefore, these two types of food were excluded from this paper, and the “healthy diet diversity” index constructed by the remaining seven types of food was used for the robustness test. According to the regression results in Table 6, column (1) shows that after gradually adding the squared power of age, the result is completely consistent with the linear regression results, which indicates that grandparents’ intergenerational care behavior will significantly improve family diet diversity by 22.9%. As can be seen in column (2), families with an intergenerational care behavior are more likely to have a healthy nutrition intake, which is also consistent with the results in Table 2. Overall, the robustness checks in Table 6 support the finding that intergenerational care contributes to better nutrition intake.

## 5. Discussion

Implementing policies to combat malnutrition, which remains a matter of concern due to the world’s effort to improve nutritional intake, is a good sign of economic growth. As China is looking for channels to innovate and structuralize its economic development through proper individual health enhancement, it is a prerequisite to scientifically define the nutritional intake determinants boosting health. Without proper nutritional intake, individual healthcare burden is likely to increase, negatively impacting productivity and economic growth and thus perpetuating the vicious cycle of poverty [44,45]. Using China Family Panel Studies (CFPS) (2010–2014), we aim to evaluate the impacts of intergenerational care behavior on nutritional intake in Chinese rural residents from the perspective of household consumption.

The herein obtained results indicated that intergenerational care has a positive and significant connection with food diversity intake based on the baseline and heterogeneous analysis results. This confirms that grandparents’ intergenerational care will increase dietary richness by one more food item. Grandparents may give support by using their saved economic or social resources as a helping tool to relieve burdens, including insufficient food, divorce, poverty, substance abuse, and mental health problems on their children. This is probably due to the traditional Chinese “parental system” and the philosophy of Confucianism influence [21]. In addition, using female labor participation and labor income as mediators in the intergenerational care behavior and nutritional intake nexus, we observed that a completely mediating effect exists. This means that intergenerational care can promote young women’s personal income, thereby promoting dietary diversity. Thus, intergenerational care can significantly increase the female labor supply, reinforcing gender equality and contributing to food security in small households, following Arpino et al. [14,46]. Consistent with Smith et al., when women earn an income in the household, family nutrition is more likely to improve than when men earn an additional income [47].

There are still many limitations in the exploration of intergenerational care and dietary diversity in this paper. Firstly, we were limited in the data used in this paper, being restricted to only that which is relatively three old years. Secondly, the current study focused on rural areas in China and did not consider urban areas’ situations. Thirdly, we consider that intergenerational care is the help of caring for grandchildren, and we did not consider the economic aspects of those services. Forthcoming studies should take note of the economic aspects in their work. Fifthly, the study focused more on the role of female householders rather than the entire householders. Future studies can take into consideration the whole household. Finally, the study did not make much referral to food waste, a possible opportunity to increase food security and reduce hunger. Future studies are recommended to expand the other geographic scope and analyze sufficient data to better investigate and understand the precise relationship between intergenerational care, food diversity, and food waste.

## 6. Conclusions and Implications

People are increasingly interested in promoting a healthy diet to reduce chronic diseases related to food worldwide [48]. In the context of aging, different from previous studies, this study presents complementary quantitative and qualitative findings based on the China Household Tracking Survey (CFPS) panel data from 2010 to 2014 and finds that intergenerational care plays an essential role in improving diet diversity through using grandparents’ human and material resources for childcare, which plays an important role in replacing childcare institutions and alleviating family economic burden. Hence, they are able to allocate more money to various food. The mediating effect method was used to explore the effects of intergenerational care behavior on diet diversity by promoting the incomes of young mothers. From the gender concept, grandparents taking care of their grandchildren can reduce the burden on young mothers, which can greatly release the labor force of young mothers and promote the labor force participation rate, improving individual incomes. Women’s higher individual income will positively influence their bargaining position within the household and family’s financial conditions, thus improving food diversity.

Additionally, the different impacts of variables on diet diversity documented in the other studies may be due to ignoring the heterogeneity of the samples [49]. Thus, to further analyze the heterogeneity of results, the whole sample was divided into different groups by region and family net income. Our heterogeneity analyses suggest that there was a difference in the effect of intergenerational care on a family diet. The regression results of the eastern region were consistent with the basic regression results, but the western region was insignificant. Furthermore, the regression results for families with a fewer level of family net income are consistent with the basic regression results but are not significant for families with higher family net income. Finally, the robustness of the model is tested by adding control variables and exchanging the model, thus also supporting the null hypothesis.

The present study has much effective theoretical and practical significance or implications. First, from the perspective of household composition and population structure, it can help identify why intergenerational care could positively affect family diet diversity. Therefore, with the advent of the three-child policy, the government should set up corresponding public child-rearing welfare institutions to ease the burden of family rearing so families can spend more energy and wealth on improving family diets. With the advent of the era of aging in our country, elderly parents caring for their children in daily life and work have played a very important influence, so the government should fully affirm the elderly contribution to families, encourage households to participate in the behavior of the internal mutual care, human capital, to give full play to the elderly family benefit maximization.

## Figures and Tables

**Table 1:** Definition and data descriptive statistics.

Variable	Definition	Mean	S.D.	N
Food diversity	Dietary diversity scores	4.907	2.022	20,314
Intergenerational care	Whether intergenerational care? Yes = 1, No = 0	0.554	0.497	17,557
Education year	0 = Illiterate; 6 = Primary school; 9 = Junior high school; 12 = High school; 15 = Secondary specialized school; 16 = Junior college or university or above	6.483	3.930	20,629
Age	Responder’s age	42.107	8.290	20,904
Marital status	Marriage or cohabitation = 1, other conditions = 0	0.972	0.165	20,902
Distance	Log (Distance to the nearest market town)	2.881	0.975	20,904
Land rent	Whether the land is rented out or not? Yes = 1, No = 0	0.080	0.271	19,108
Family size	Total household number	4.674	1.646	20,904
Child number	Total family’s children number	1.779	0.803	20,904
Grandparents’ education	Years of education by the grandparents	5.214	6.026	18,418
Income	Log (family net income)	10.147	0.932	19,542

S.D. = Standard Deviation and N = number of sample size.

**Table 2:** The impact of intergenerational care on food diversity for the unbalanced panel data estimated results.

Variable	(1) FE	(2) RE	(3) 2SLS
Intergenerational care	0.224 ***	0.075 **	3.101 ***
	(0.055)	(0.038)	(0.604)
Education year	0.065 ***	0.074 ***	0.057 ***
	(0.012)	(0.005)	(0.019)
Age	0.200 ***	0.005 ***	0.147 ***
	(0.013)	(0.003)	(0.024)
Marital status	0.219	0.163 *	0.478
	(0.282)	(0.115)	(0.435)
Distance	−0.005	−0.170 ***	−0.053
	(0.030)	(0.018)	(0.054)
Land rent	0.025	0.348 ***	−0.180
	(0.092)	(0.060)	(0.151)
Family size	−0.044	−0.057 ***	−0.069
	(0.035)	(0.013)	(0.052)
Income	0.121 ***	0.445 ***	0.159 ***
	(0.029)	(0.020)	(0.047)
Grandparents’ education year	0.022 *** (0.006)	0.025 (0.003)	0.010 (0.011)
Child numbers	0.062 (0.094)	−0.072 (0.026)	−0.028 (0.155)
Constant	−5.207 *** (0.580)	0.212 (0.234)	
R-squared F	0.079		−0.373 79
Observation	12,595	12,595	5624

Robust standard errors are in parentheses; * *p* < 0.10, ** *p* < 0.05, *** *p* < 0.01.

**Table 3:** Mediating effect analysis of female labor participation and labor income estimated result.

Variable		Coefficient	Standard Error	Z	*p*	95% Confidence Interval	
Female income	Indirect effect	0.0127	0.0059	2.18	0.030	0.001	0.024
	direct effect	0.126	0.078	1.61	0.107	−0.027	0.279

**Table 4:** The impact of intergenerational care on food diversity by regional heterogeneity indicators among three Chinese areas (eastern, central, and western) estimated result.

Variable	Eastern Rural Areas	Central Rural Areas	Western Rural Areas
Intergenerational care	0.085	0.164 *	0.468 ***
	(0.090)	(0.098)	(0.105)
constant	−8.288 ** (1.012)	−1.145 (1.025)	−5.360 * (1.091)
R-squared	0.130	0.033	0.095
Control variables	Yes	Yes	Yes
Observation	4473	3727	3566

Note: Robust standard errors are in parentheses; * *p* < 0.1, ** *p* < 0.05, *** *p* < 0.01.

**Table 5:** The impact of intergenerational care on food diversity by income heterogeneity indicators among rural families (lower income family and higher income family) estimated result.

Variable	Lower Family Net Income	Higher Family Net Income
Intergenerational care	0.306 ***	0.143
	(0.075)	(0.136)
Constant	−6.715 (0.882)	−2.603 (1.905)
R-squared	0.092	0.051
Control variables	Yes	Yes
Observation	8321	4274

Robust standard errors are in parentheses; *** *p* < 0.01.

**Table 6:** Robustness check of the baseline estimated result.

Variable	Food Diversity	Healthy Food Diversity
intergenerational care	0.229 ***	0.162 ***
	(0.055)	(0.043)
Constant	−0.258 (1.298)	−5.379 *** (0.478)
R-squared	0.083	0.113
Control variables	Yes	Yes
Observation	12,595	12,595

Note: Robust standard errors are in parentheses; *** *p* < 0.01.

## Data Availability

The data were released to the researchers without access to any personal data. Data access link: http://www.isss.pku.edu.cn/cfps/ (accessed on 4 June 2022).

## References

[1] Bei Y., Yang T., Xiao J. (2018). Cardiovascular medicine in China: What can we do to achieve the Healthy China 2030 plan?. BMC Med..

[2] Romieu I., Dossus L., Barquera S., Blottière H.M., Franks P.W., Gunter M., Hwalla N., Hursting S.D., Leitzmann M., Margetts B. (2017). Energy balance and obesity: What are the main drivers?. Cancer Causes Control.

[3] Ren Y., Zhang Y., Loy J.-P., Glauben T. (2018). Food consumption among income classes and its response to changes in income distribution in rural China. China Agric. Econ. Rev..

[4] Zanello G., Shankar B., Poole N. (2019). Buy or make? Agricultural production diversity, markets and dietary diversity in Afghanistan. Food Policy.

[5] Wan Y., Hu H., Hu W. (2022). Agricultural production structure, market conditions and farmers’ nutritional intake in rural China. J. Integr. Agric..

[6] Ma B., Jin X. (2022). Does Internet Use Connect Us to a Healthy Diet? Evidence from Rural China. Nutrients.

[7] Wang Y., Wen X., Zhu Y., Xiong Y., Liu X. (2022). Chinese Residents’ Healthy Eating Intentions and Behaviors: Based on an Extended Health Belief Model. Int. J. Environ. Res. Public Health.

[8] Ren Y., Castro Campos B., Peng Y., Glauben T. (2021). Nutrition transition with accelerating urbanization? Empirical evidence from rural China. Nutrients.

[9] Galiè A., Teufel N., Girard A.W., Baltenweck I., Dominguez-Salas P., Price M.J., Jones R., Lukuyu B., Korir L., Raskind I. (2019). Women’s empowerment, food security and nutrition of pastoral communities in Tanzania. Glob. Food Secur..

[10] Gao Y., Zheng Z., Henneberry S.R. (2020). Is nutritional status associated with income growth? Evidence from Chinese adults. China Agric. Econ. Rev..

[11] You J. (2013). The role of microcredit in older children’s nutrition: Quasi-experimental evidence from rural China. Food Policy.

[12] Huang Y., Tian X. (2019). Food accessibility, diversity of agricultural production and dietary pattern in rural China. Food Policy.

[13] Fard N.A., Morales G.D.F., Mejova Y., Schifanella R. (2021). On the interplay between educational attainment and nutrition: A spatially-aware perspective. EPJ Data Sci..

[14] Sekabira H., Qaim M. (2017). Can mobile phones improve gender equality and nutrition? Panel data evidence from farm households in Uganda. Food Policy.

[15] Redman B.J. (1980). The impact of women’s time allocation on expenditure for meals away from home and prepared foods. Am. J. Agric. Econ..

[16] Liu H., Wahl T.I., Seale Jr J.L., Bai J. (2015). Household composition, income, and food-away-from-home expenditure in urban China. Food Policy.

[17] Bongaarts J. (2006). United nations department of economic and social affairs, population division world mortality report 2005. Popul. Dev. Rev..

[18] Whyte M.K. (2004). Filial Obligations in Chinese Families: Paradoxes of Modernization.

[19] Chen F., Liu G. (2012). The health implications of grandparents caring for grandchildren in China. J. Gerontol. Ser. B Psychol. Sci. Soc. Sci..

[20] Chen F., Liu G., Mair C.A. (2011). Intergenerational ties in context: Grandparents caring for grandchildren in China. Soc. Forces.

[21] Burnette D., Sun J., Sun F. (2013). A comparative review of grandparent care of children in the US and China. Ageing Int..

[22] Fuller-Thomson E., Minkler M., Driver D. (1997). A profile of grandparents raising grandchildren in the United States. Gerontologist.

[23] Croll E.J. (2006). The intergenerational contract in the changing Asian family. Oxf. Dev. Stud..

[24] Hirshorn B.A. (1998). Grandparents as caregivers. Handbook on Grandparenthood.

[25] Presser H.B. (1989). Some economic complexities of child care provided by grandmothers. J. Marriage Fam..

[26] Guzman L. (2004). Grandma and Grandpa Taking Care of the Kids: Patterns of Involvement. Child Trends Research Brief.

[27] Colen L., Melo P., Abdul-Salam Y., Roberts D., Mary S., Paloma S.G.Y. (2018). Income elasticities for food, calories and nutrients across Africa: A meta-analysis. Food Policy.

[28] Koppmair S., Kassie M., Qaim M. (2017). Farm production, market access and dietary diversity in Malawi. Public Health Nutr..

[29] Codjoe S.N.A., Okutu D., Abu M. (2016). Urban household characteristics and dietary diversity: An analysis of food security in Accra, Ghana. Food Nutr. Bull..

[30] Ji W., Du J., Li X., Liu Y., Liang A. (2020). Incidence of eating problems and related factors in children aged 1–6 years. Zhonghua Liu Xing Bing Xue Za Zhi = Zhonghua Liuxingbingxue Zazhi.

[31] Chinnadurai M., Karunakaran K., Chandrasekaran M., Balasubramanian R., Umanath M. (2016). Examining Linkage between Dietary Pattern and Crop Diversification: An Evidence from Tamil Nadu. Agric. Econ. Res. Rev..

[32] Jones A.D., Shrinivas A., Bezner-Kerr R. (2014). Farm production diversity is associated with greater household dietary diversity in Malawi: Findings from nationally representative data. Food Policy.

[33] Ruel M.T. (2003). Operationalizing dietary diversity: A review of measurement issues and research priorities. J. Nutr..

[34] Drescher L., Thiele S., Roosen J., Mensink G.B. (2009). Consumer demand for healthy eating considering diversity–an economic approach for German individuals. Int. J. Consum. Stud..

[35] Morseth M.S., Grewal N.K., Kaasa I.S., Hatloy A., Barikmo I., Henjum S. (2017). Dietary diversity is related to socioeconomic status among adult Saharawi refugees living in Algeria. BMC Public Health.

[36] Tafreschi D. (2015). The income body weight gradients in the developing economy of China. Econ. Hum. Biol..

[37] Wen Z., Ye B. (2014). Analyses of mediating effects: The development of methods and models. Adv. Psychol. Sci..

[38] Chegere M.J., Stage J. (2020). Agricultural production diversity, dietary diversity and nutritional status: Panel data evidence from Tanzania. World Dev..

[39] Ren Y., Li H., Wang X. (2019). Family income and nutrition-related health: Evidence from food consumption in China. Soc. Sci. Med..

[40] MacKinnon D.P., Lockwood C.M., Hoffman J.M., West S.G., Sheets V. (2002). A comparison of methods to test mediation and other intervening variable effects. Psychol. Methods.

[41] Preacher K.J., Hayes A.F. (2004). SPSS and SAS procedures for estimating indirect effects in simple mediation models. Behav. Res. Methods Instrum. Comput..

[42] Zhao M., Konishi Y., Glewwe P. (2013). Does information on health status lead to a healthier lifestyle? Evidence from China on the effect of hypertension diagnosis on food consumption. J. Health Econ..

[43] Sun J.-J., Zhang H. (2013). The situation and influencing factors of Chinese older people taking care of their grandchildren. Popul. Econ..

[44] Fogel R.W. (2004). Health, nutrition, and economic growth. Econ. Dev. Cult. Chang..

[45] Sarkar S. (2015). Consumption pattern and determinants of nutritional intake among rural households of West Bengal, India. J. Settl. Spat. Plan..

[46] Arpino B., Pronzato C.D., Tavares L.P. (2014). The effect of grandparental support on mothers’ labour market participation: An instrumental variable approach. Eur. J. Popul..

[47] Smith L.C., Ramakrishnan U., Ndiaye A., Haddad L., Martorell R. (2003). The importance of Women’s status for child nutrition in developing countries: International food policy research institute (IFPRI) research report abstract 131. Food Nutr. Bull..

[48] Sun Y., Dong D., Ding Y. (2021). The impact of dietary knowledge on health: Evidence from the China Health and nutrition survey. Int. J. Environ. Res. Public Health.

[49] O’brien G., Davies M. (2007). Nutrition knowledge and body mass index. Health Educ. Res..

